# Detecting positive selection in the genome

**DOI:** 10.1186/s12915-017-0434-y

**Published:** 2017-10-30

**Authors:** Tom R. Booker, Benjamin C. Jackson, Peter D. Keightley

**Affiliations:** Institute of Evolutionary Biology, University of Edinburgh, Edinburgh, EH9 3FL UK

## Abstract

Population geneticists have long sought to understand the contribution of natural selection to molecular evolution. A variety of approaches have been proposed that use population genetics theory to quantify the rate and strength of positive selection acting in a species’ genome. In this review we discuss methods that use patterns of between-species nucleotide divergence and within-species diversity to estimate positive selection parameters from population genomic data. We also discuss recently proposed methods to detect positive selection from a population’s haplotype structure. The application of these tests has resulted in the detection of pervasive adaptive molecular evolution in multiple species.

## Neutral theory and the extent of selection

The extent to which positive selection contributes to molecular evolution has been a long-standing question in evolutionary genetics. The classic paradigm in modern evolutionary genetics has been the neutral theory, which contends that the vast majority of molecular changes are a consequence of genetic drift, positive selection playing only a minor role [1]. However, it is becoming increasingly clear that natural selection, both positive and negative, is pervasive in many genomes, to such an extent that negative selection has been proposed as a null model for explaining variation in levels of genetic diversity across the genome [2]. Indeed, the question currently asked by researchers is no longer ‘is positive selection present?’ but instead ‘how frequent and strong is positive selection?’. Fittingly, then, a number of different approaches have been proposed to quantify the frequency and strength of positive selection using population genetic (and genomic) approaches.

**Fig. 1.**
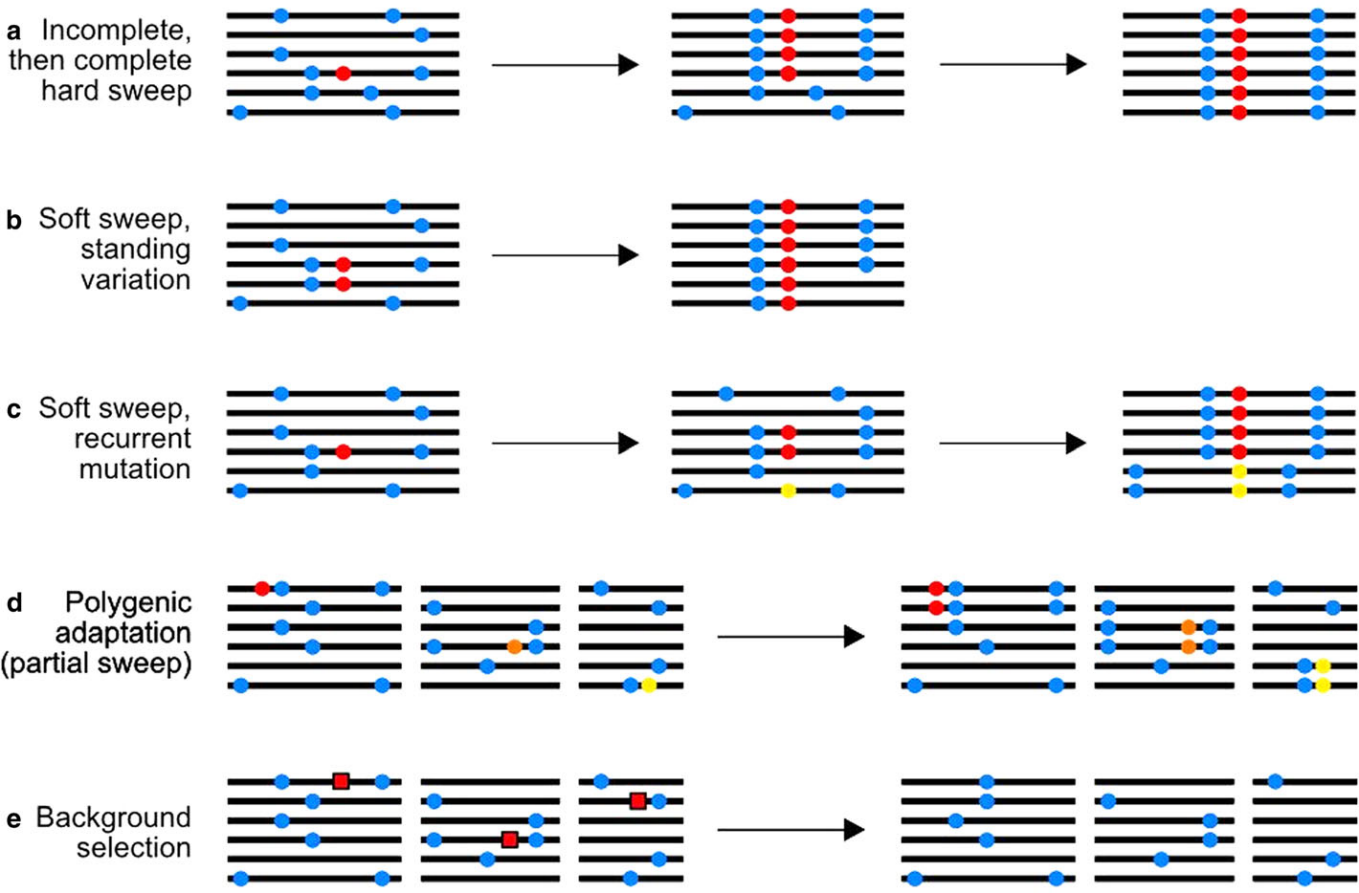
Selective sweeps and background selection. Maynard Smith and Haigh [79] showed that as an advantageous mutation rises in frequency it drags with it linked neutral polymorphisms. With increasing genetic distance from the selected site, the effect is reduced, resulting in troughs in genetic diversity in surrounding regions. **a** Hard/classic sweeps - the most well-studied model of sweeps. A new advantageous mutation rapidly increases in frequency to eventual fixation. As it sweeps, the adaptive allele carries with it a portion of the haplotype on which it arose, reducing levels of neutral diversity in the surrounding area [27, 79]. **b**-**c** Soft sweeps - a neutral allele segregating in a population may become favoured (due, for example, to a change in the environment). **b** The segregating allele may be associated with multiple haplotypes, and as it rises in frequency, so do the multiple haplotypes. **c** A similar process, also termed a soft sweep, can occur if an advantageous mutation arises by multiple, distinct mutation events. See [66] for a thorough review on soft sweep models. **d** Incomplete/partial sweeps - If an advantageous allele increases in frequency, but does not reach fixation, there will still be some loss of linked neutral diversity. In this review, we use the term incomplete sweeps to describe sweeps that are polymorphic at the time of sampling, but may (or may not) eventually reach fixation **a**. The term partial sweep describes the situation wherein a sweeping allele becomes effectively neutral at a certain frequency in its trajectory **d**. The magnitude of both processes’ effects on linked neutral diversity depends on the frequency reached by the sweeping allele when selection is ‘turned off’ or on the time of sampling [33]. Partial sweeps may be common in cases of adaptation involving selection on quantitative traits [67]. **e** Background selection - as natural selection purges deleterious mutations, neutral alleles linked to the selected locus are also lost. The process of background selection is qualitatively similar to recurrent selective sweeps, since both processes reduce local genetic diversity [80] and skew the SFS towards rare variants [81, 82]. Models of background selection envisage a neutral site linked to many functional sites at different distances, such that the effects of selection at many sites accumulate to reduce diversity [83, 84]. Blue circles represent neutral alleles, red, yellow and orange circles represent advantageous alleles, and red squares represent deleterious alleles

The purpose of this review is to describe the different lines of evidence that have been used to determine the frequency and strength of positive selection in multiple species. We will start by discussing the McDonald-Kreitman test [3] and its extensions, which have been used to quantify the frequency of adaptive molecular evolution acting directly on protein-coding genes. We then discuss how predictions of selective sweep models (Fig. 1) can be used to estimate the parameters of positive selection indirectly, using variability at linked neutral sites. Finally, we describe how recent results from large-scale genomic datasets have challenged the bases of these methods. Note, we will not focus on the many methods to identify individual adaptive events or genome scans to detect local adaptation (for a review, see [4]), nor will we discuss experimental evolution (for reviews, see [5] and [6]).

## Quantifying the frequency of positive selection—the McDonald-Kreitman test

Some of the strongest evidence for adaptive molecular evolution has come from application of the McDonald-Kreitman (MK) test [3] and methods based on it. Testing for evidence of positive selection requires a suitable null hypothesis. Under the neutral hypothesis of molecular evolution, differences accumulate by genetic drift, positive selection playing only a minor role [1]. The MK test can be used to test for positive selection by comparing within-species nucleotide diversity and between-species nucleotide divergence for sites subject to natural selection and sites assumed to be evolving neutrally. Most studies have analyzed nonsynonymous sites of protein-coding genes, using synonymous sites as a neutral reference. We will focus on such analyses here, although the MK test has also been applied to a variety of non-coding genomic elements in several species. If synonymous mutations evolve neutrally and nonsynonymous mutations are either neutral or are strongly deleterious, the ratio of the number of nonsynonymous to synonymous polymorphisms for a gene (*P*_*n*_/*P*_*s*_) is expected to be equal to the ratio of nonsynonymous to synonymous divergence (*D*_*n*_/*D*_*s*_) (although it should be noted that measures of polymorphism and divergence are not entirely independent). Strongly positively selected mutations, however, will inflate *D*_*n*_, while contributing negligibly to *P*_*n*_ (Table 1).

**Table 1:** MK table for the *Adh* gene [3] showing numbers of fixed differences and polymorphic sites between and within *D. melanogaster, D. simulans* and *D. yakuba*

	Differences (*D*)	Polymorphism (*P*)
Nonsynonymous	7	2
Synonymous	17	42

Note that the ratio of fixed nonsynonymous to synonymous differences (7/17) is substantially higher than the ratio of nonsynonymous to synonymous polymorphisms (2/42), indicating that some amino acid differences are adaptive

The MK test ratios allow estimation of the fraction of nonsynonymous differences, α, driven to fixation by position selection for a set of genes or other class of sites [7]:

$$ \alpha =1-\frac{D_S{P}_n}{D_n{P}_s} $$

**Fig. 2.**
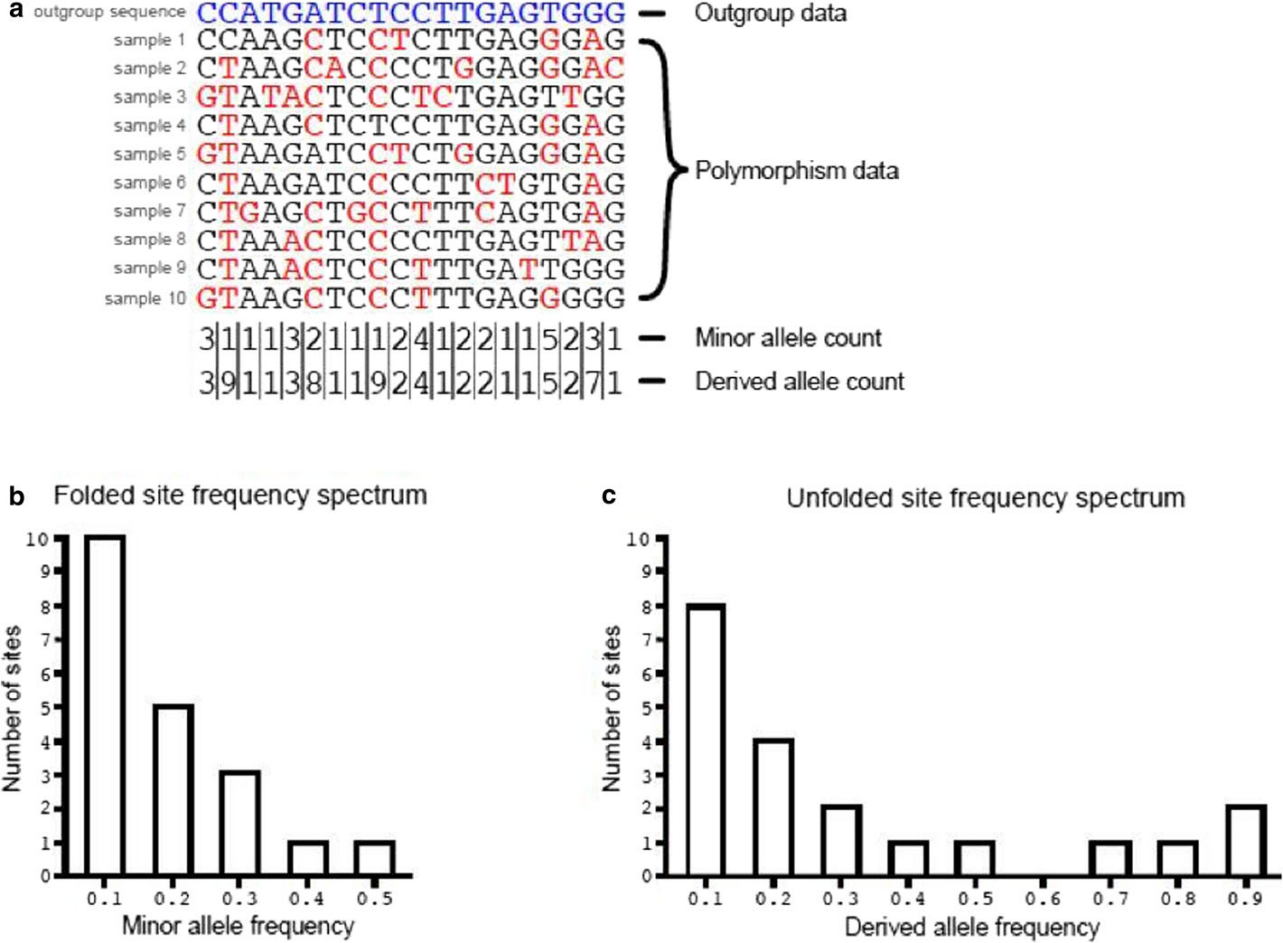
The site frequency spectrum. The numbers of variants segregating at different frequencies in a population can be summarized as the site frequency spectrum (SFS). Consider the ten chromosome samples shown in **a**. Observations of a particular minor allele frequency are used to populate the folded SFS **b**. ‘Unfolding’ the SFS requires knowledge of whether alleles are ancestral or derived. Aligning sequenced data to an outgroup (the blue nucleotides in **a**) allows the inference of ancestral and derived states for polymorphic and diverged sites, by maximum parsimony. However, the parsimony approach makes a number of biologically unrealistic assumptions; for example, that there have been no mutations in the lineage leading to the outgroup. Because of these, a number of alternative approaches have been proposed that have been shown to be more accurate than parsimony (e.g. [15]). Various evolutionary processes can alter the SFS, including directional and balancing selection, gene conversion, population size change and migration. For example, purifying selection prevents harmful variants from rising in frequency, resulting in a skew in the SFS towards rare variants. Multiple statistics have been proposed to summarize both the folded and unfolded SFS, and these can shed light on the evolutionary process (reviewed in [4])

A weakness of this approach is that it assumes the strict neutral model, where deleterious nonsynonymous mutations can be frequent, but are assumed to be strongly selected against, such that they contribute negligibly to polymorphism and divergence. If there are slightly deleterious mutations, these will tend to inflate *P*_*n*_ while not becoming fixed. This reduces the power to detect adaptive evolution for a given gene and potentially downwardly biases estimates of α for a group of genes. Omitting low frequency variants preferentially removes slightly deleterious mutations and can potentially reduce this bias [8, 9], but the result is sensitive to the arbitrary cut-off value chosen. More recently, approaches for estimating α have been developed that use the spectrum of allele frequencies [10–13], explicitly modeling the contribution of deleterious mutations to polymorphism and divergence. Within all of these approaches, the distribution of fitness effects (DFE) of nonsynonymous mutations is estimated, based on the relative levels of nonsynonymous versus synonymous polymorphism and the properties of the frequency distribution of numbers of allele copies present at segregating sites (the ‘site frequency spectrum’ (SFS); Fig. 2).

Various models for the DFE have been assumed in these analyses, a common one being the gamma distribution. The estimated parameters of the DFE are then used to calculate the expected number of nonsynonymous differences between the species pair; the difference between the observed and the expected divergence is attributed to positively selected mutations and used to estimate α [14] (Box 1). It is possible to base inferences on the unfolded or folded SFS (Fig. 2); in the former case, polymorphisms need to be polarised using outgroup species, and it is then feasible to include advantageous mutations within the analysis [12]. It is also possible to base inferences solely on standing polymorphism, that is, to ignore the between-species divergence altogether [13, 15]. With all these different flavors of the basic method, recent demographic changes, altering the shape of both the synonymous and nonsynonymous SFSs compared to that expected under the neutral model, are incorporated in the analysis. Correcting for demographic change by allowing changes in effective population size appears to substantially correct for other genome-wide processes that distort the SFSs, such as background selection [16].

## Box 1 Calculation of α and *ω*_*a*_ using estimates of the distribution of fitness effects of new mutations

Assume we are focusing on the evolution of protein-coding genes between two species, and that we have polymorphism data for a focal species. The amino acid divergence between the species (*D*_*n*_) is the sum of the divergence attributable to positively selected mutations (*D*_*a*_) and that attributable to the fixation of neutral and slightly deleterious mutations (*D*_*na*_):

$$ {D}_n={D}_a+{D}_{na} $$

The amino acid divergence can be estimated directly from the sequence data of the two species. Methods such as DFE-alpha [11] infer *D*_*na*_ by calculating the average fixation probability of a deleterious mutation—based on the distribution of fitness effects of new deleterious mutations—estimated from the information contained in the folded nonsynonymous and synonymous site frequency spectra (Fig. 2) of the focal species. The adaptive divergence is then *D*_*a*_ = *D*_*n*_ − *D*_*na.*_ The estimated proportion of amino acid substitutions driven to fixation by positive selection (α) is the ratio of the adaptive divergence (*D*_*a*_) and the amino acid divergence (*D*_*n*_):

$$ \upalpha ={D}_a/{D}_n $$

An alternative and potentially more informative estimator of the frequency of adaptive molecular evolution is *ω*_*a*_, the ratio of the adaptive divergence and the synonymous divergence:

$$ {\omega}_a={D}_a/{D}_s $$

Galtier [13] proposed a complementary statistic, *ω*_*na*_, which gives an estimate of the rate of non-adaptive amino acid substitutions.

## Empirical findings from applying the MK test and its derivatives

While initial results from the application of these approaches were somewhat confusing, a more consistent picture emerged as larger data sets became available. Initial results indicated that adaptive protein evolution is widespread in *Drosophila*, with α values typically as high as 40% [17], whereas estimates for humans were generally substantially lower and in some cases nonsignificantly different from zero [17].

The frequency of adaptive substitution is expected to be higher in populations of large effective size, *N*_*e*_, since the probability of fixation of a newly arising advantageous mutation increases with *N*_*e*_ [18], and more advantageous mutations appear in large populations. However, α is not simply a function of the rate of fixation of advantageous mutations, since the overall rate of substitution (the denominator used in the calculation of α) includes the rate of fixation of deleterious mutations (Box 1), and these are expected to fix less frequently in large populations. This implies that α should increase with *N*_*e*_, even if the rate of fixation of advantageous mutations does not change. Campos et al. [19] observed a positive correlation between α and the rate of recombination for protein-coding genes in the *Drosophila melanogaster* genome. Since *N*_*e*_ for a genomic region is positively related to the rate of recombination [20], increased rates of fixation of advantageous mutations and decreased rates of fixation of deleterious mutations are expected in high recombination regions. Campos et al. also observed that the rate of recombination is positively correlated with ω_*a*_, the estimated rate of advantageous substitution relative to the rate of neutral substitution (Box 1), suggesting that beneficial substitutions increase with increasing recombination rate, perhaps due to decreasing interference between selected loci [21].

Similarly, a positive correlation between the *N*_*e*_ for a species and ω_*a*_ was observed by Gossmann et al. [22] in an analysis of protein-coding genes from 13 eukaryotic species pairs. Evidence from a much larger study [13], however, does not support a relationship between *N*_*e*_ and the rate of adaptive molecular evolution. Galtier [13] studied protein-coding genes in 44 metazoan species pairs to investigate the relationships between the rate of adaptive evolution (measured using α and ω_*a*_) and *N*_*e*_. There was a positive relationship between α and *N*_*e*_, but a negative relationship between the estimated rate of fixation of deleterious mutations (ω_*na*_) and *N*_*e*_. However, ω_*a*_ was nonsignificantly correlated with *N*_*e*_, implying that the positive correlation between *N*_*e*_ and α is driven by variation in the fixation rate of deleterious mutations. This result also implies that adaptation of protein-coding genes may not be limited by the supply of new mutations.

## Are most amino acid substitutions adaptive?

A notable conclusion from Galtier’s study is that average α exceeds 50%, implying that most amino acid substitutions are adaptive in many species. Primates, notably hominids, are an exception, tending to have lower α, presumably because of their small effective population sizes, leading to the accumulation of slightly deleterious amino acid mutations. Taken at face value, Galtier’s study is, therefore, a strong challenge to the neutral hypothesis of molecular evolution, as it suggests that a large proportion of amino acid changes resulted from positive selection in a variety of species. There are, however, several caveats. First, if selection is operating in the reference class of sites (in the case of protein-coding genes, selection on codon usage operating on synonymous sites), upwardly biased estimates of α are expected [23], and this kind of selection is most prevalent in species with large *N*_*e*_. Second, Fay [24] highlights a number of difficulties with the MK-based approach, including local adaptation and epistasis among deleterious mutations, both of which could inflate values of α. Finally, Galtier included ‘mirror species pairs’ where polymorphism data were available for both species of the pair, and two estimates of α and ω_*a*_ could therefore be calculated. While estimates of these quantities were mostly in reasonable agreement, one mirror species pair from an earlier study of ours (the house mouse and brown rat) produced strikingly different estimates: α = +0.32 if polymorphism data for mice are analyzed and α = −0.29 if data from rats are analyzed [25]. The negative estimate for rats was attributed to a population bottleneck in the brown rat, increasing the frequency of slightly deleterious amino acid mutations in current rat populations. Nucleotide divergence between mice and rats accumulated over a much longer time-scale, however, and was presumably largely unaffected by this bottleneck. Similar results have been found for several plant species, where estimates of α are for the most part close to zero [26], and in some cases significantly less than zero. These examples highlight a fundamental problem with the MK-based approach—within-species nucleotide diversity and between-species divergence can be decoupled from one another by ancient demographic events not captured by current polymorphism data, potentially undermining the ability to estimate the prevalence of adaptive molecular evolution.

## Using models of selective sweeps to estimate positive selection parameters

If adaptive substitutions are common, selection is expected to leave footprints in genetic diversity at linked sites. In particular, as a positively selected mutation increases in frequency, it tends to reduce diversity at linked neutral loci. Theoretical analyses of this process, termed a selective sweep (Fig. 1), have shown that the reduction in diversity at a linked neutral locus depends on the ratio of the strength of positive selection to the recombination rate [27]. Thus, comparing diversity at multiple neutral loci linked to selected regions, in principle, should provide an indirect means for estimating the average rate and strength of positive selection in the genome.

If a population experiences recurrent selective sweeps, several patterns are predicted by theory. Under recurrent selective sweeps, levels of genetic diversity are expected to be lower i) in regions of the genome with restricted recombination, ii) in regions experiencing many sweeps and iii) in the genomic regions surrounding the targets of selection themselves. Each of these predictions has been met in empirical studies, and each has been used to estimate parameters of positive selection using models of hard selective sweeps.

### The correlation between diversity and the rate of recombination

In the late 1980s, evidence began to emerge suggesting that genetic polymorphism is reduced in genomic regions that experience restricted crossing-over [28, 29]. Soon after, Begun and Aquadro [30] showed that there is a positive correlation between nucleotide diversity and the rate of crossing-over in *D. melanogaster*, a pattern subsequently observed in other eukaryotic species [31]. Begun and Aquadro pointed out that the correlation is qualitatively consistent with the action of recurrent selective sweeps. Wiehe and Stephan [32] formulated expressions, based on the correlation between nucleotide diversity and the rate of recombination, to estimate the compound parameter for the intensity of selection *λ2N*_*e*_ *s*, where *λ* is the rate of sweeps per base pair per generation, *N*_*e*_ is the effective population size and *s* is the selection coefficient (the reduction in relative fitness experienced by homozygotes), assuming semi-dominance. They applied their method to the data of Begun and Aquadro [30], estimating *λ2N*_*e*_ *s* = 5.37 × 10^−8^, but their method could not disentangle the individual parameters. More recently, Coop and Ralph [33] performed a similar analysis in *D. melanogaster* to explore the effects of partial sweeps on parameter estimates. They showed that when partial sweeps are common, the rate of adaptive evolution is underestimated if the hard sweep model is assumed.

The correlation between diversity and recombination observed by Begun and Aquadro [30] can also be explained by background selection, the reduction in neutral diversity caused by the removal of linked deleterious mutations (Fig. 1) [34]. The correlation between neutral diversity and the recombination rate predicted by background selection is quantitatively similar to that observed in *D. melanogaster* [35]. Indeed, recent studies suggest that background selection is a major determinant of nucleotide diversity variation at broad scales (>100 kbp) in humans [36] and *D. melanogaster* [2, 37]. It is clear, then, that background selection is a key confounding factor when attempting to make inferences about positive selection from diversity patterns.

### Correlation between neutral diversity and non-neutral divergence

Under a model of recurrent sweeps, there should be a negative correlation between nucleotide divergence at selected sites and diversity at linked neutral sites. This is because rapidly evolving regions of the genome will experience more sweeps, which will reduce levels of linked neutral diversity more than slowly evolving regions. The relationship between neutral diversity and selected divergence should therefore carry information on the rate and strength of selective sweeps.

The abovementioned correlation was first described by Andolfatto [38] for the X chromosome of *D. melanogaster* using synonymous site diversity and non-synonymous divergence, and has been subsequently reported in other *Drosophila* species [39]. Using the correlation, Andolfatto [38] estimated the compound parameter for the intensity of selection *λ2N*_*e*_ *s* = 3 × 10^−8^ for the X chromosome in *D. melanogaster* (similar to the value obtained based on the correlation of synonymous site diversity and recombination rate [32]; see above). Using an estimate of α obtained from an MK-based analysis, Andolfatto [38] decomposed *λ2N*_*e*_ *s* into its constituent parameters and found that advantageous mutations in the protein-coding genes of *D. melanogaster* are moderately weakly selected but relatively frequent. In a similar study, Macpherson et al. [40] examined the correlation between mean neutral diversity and selected (nonsynonymous) divergence in *Drosophila simulans,* and estimated *λ2N*_*e*_ *s* to be *~* 10^−7^. However, they used a model that also included the heterogeneity in levels of diversity, which is related to the rate and strength of sweeps in a different way to the mean, allowing them to obtain estimates of the *λ* and *s* parameters by regression. Although estimates of the compound parameter *λ2N*_*e*_ *s* are similar between the two studies, the estimated rate and fitness effect parameters were quite different, Macpherson et al. [40] estimating that advantageous mutations are relatively rare and have large fitness effects. The discrepancies between the studies may be due to differences in biology between *D. melanogaster* and *D. simulans*, or may reflect differences in methodology. For example, if the majority of adaptive substitutions are driven by weakly selected sweeps, which will leave a relatively small signal in levels of polymorphism, the MK-based method may more sensitively detect them, perhaps explaining the higher rate of sweeps inferred by Andolfatto [38]. On the other hand, strongly selected sweeps will leave a larger footprint in levels of diversity, so will be more readily detected using the approach of Macpherson et al. [40], perhaps explaining why they inferred a lower overall rate of sweeps, with higher selection coefficients (for a full description, see [41]). In both cases, inferences based on variation in polymorphism may reflect processes other than the fixation of adaptive alleles that have gone to fixation, such as partial sweeps and background selection, since these will affect patterns of diversity but not necessarily divergence. Recently, Campos et al. [42] estimated positive selection parameters from the correlation between synonymous site diversity and non-synonymous divergence across the entire *D. melanogaster* genome in the presence of both background selection and gene conversion. Their parameter estimates suggest that strongly selected advantageous mutations are relatively infrequent, making up ~ 0.02% of all new mutations at nonsynonymous sites.

In summary, analysis of the correlation between neutral diversity and putatively selected divergence has suggested that advantageous mutations in *Drosophila* are either relatively frequent, but weakly selected, or rare and strongly selected. Obviously, assuming that all advantageous mutations that occur in a genome belong to a single class of fitness effects is an oversimplification of what is likely to be a complex distribution. It may be that the discrepancy between the above studies comes about because they are capturing different parts of the distribution of fitness effects for positively selected mutations. This is corroborated by recent work described below.

### Patterns of diversity around the targets of selection

An individual hard selective sweep is expected to leave a trough in genetic diversity around the selected site. If a large proportion of amino acid substitutions are adaptive, as suggested by MK-type analyses (see above), collating patterns of diversity around all substitutions of a given type should reveal a trough in diversity. Such a pattern is not expected around a ‘control’ class of sites, such as synonymous sites. This test, proposed by Sattath et al. [43], was first applied to *D. simulans*, and the above pattern was found. By fitting a hard sweep model to the shape of the diversity trough, they estimated α values of 5 and 13%, depending on whether one or two classes of beneficial mutational effects were fitted. Note that their estimates of α are substantially lower than those obtained using MK-based methods for *D. melanogaster* [38]. Sattath et al. [43] suggested that modes of selection other than hard sweeps may help explain this discrepancy. However, even when modelling two classes of beneficial mutations, they found that amino acid substitutions are driven by relatively strongly adaptive mutations (*s ~* 0.5% and s ~ 0.01%). Their estimates of the selection strength are therefore in broad agreement with the estimate of *s* ~ 1% obtained by Macpherson et al. [40], based on the correlation between synonymous diversity and non-synonymous divergence in *D. simulans*. The results from the Sattath et al. [43] analysis are consistent with the hypothesis that adaptation in protein-coding genes is fairly frequent and driven by strong, hard sweeps.

The Sattath test has since been applied in a variety of organisms, including humans [44], house mice [45], *Capsella grandiflora* [46] and maize [47]. In all but *C. grandiflora*, researchers have found no difference in patterns of diversity around selected and neutral substitutions. These results have been interpreted as evidence that hard sweeps were rare in the recent history of both humans [44] and maize [47]. However, Enard et al. [48] pointed out that the Sattath test will be underpowered if there is large variation in levels of functional constraint in the genome. Indeed, through their analyses Enard et al. [48] found evidence for frequent adaptive substitutions in humans, particularly in regulatory sequence. To address the issues raised by Enard et al. [48], Beissinger et al. [47] applied the Sattath test to substitutions in maize genes with the highest and lowest levels of functional constraint separately, but still found no difference in diversity pattern, suggesting either that hard sweeps have been rare in that species or that there is another confounding factor.

One possible explanation is that the species in which the Sattath test did/did not detect hard sweeps have distinct patterns of linkage disequilibrium (LD). LD decays to background levels within hundreds of base-pairs in *C. grandiflora* [49] and *Drosophila* [50], whereas in humans, maize and wild house mice it decays over distances closer to 10,000 bp [25, 51, 52]. It may be, therefore, that the Sattath test is only applicable when there is relatively short-range LD, such that the patterns of diversity around selected substitutions are decoupled from the patterns of diversity around neutral substitutions. If this were the case, interpreting the similarity in troughs of diversity around selected and neutral substitutions as evidence for a paucity of hard selective sweeps may not be justified in organisms where LD decays over distances of a similar order of magnitude as the width of the diversity troughs themselves.

## Fitting genome-wide variation in nucleotide diversity and divergence

Methods for estimating the rate and strength of positive selection in the genome employ various combinations of nucleotide diversity, divergence, recombination rates and estimates of background selection effects as summary statistics, averaged over many regions of the genome. Recently, Elyashiv et al. [53] developed a method that fits a model of hard sweeps and background selection to genome-wide variation in nucleotide diversity and divergence (at both selected and neutral sites). In *D. melanogaster,* they showed that hard sweeps can explain a large amount of genome-wide variation in genetic diversity. For nonsynonymous sites, they found that α = 4.1% for strongly selected mutations (*s* ≥ 0.03%) and α = 36.3% for weakly selected mutations (*s* ~ 0.0003%), summing to α = 40.4%, which is similar to the estimate obtained using the MK test [38]. Their results suggest that accounting for weakly selected mutations may help reconcile the discrepancy between MK-based estimates of the rate and strength of selection and parameters estimated from sweep model predictions, described above.

Elyashiv et al. [53] showed that the variation in nucleotide diversity expected under a model combining the diversity-reducing effects of hard sweeps and background selection is capable of explaining a large amount of the variation in diversity across the genome, further demonstrating that the action of natural selection is likely to be pervasive, at least in *D. melanogaster*. However, several points need to be considered regarding their results. Firstly, the strength of selection on the weakly selected class of beneficial mutations in Elyashiv et al.’s study may be too weak (assuming *N*_*e*_ = 10^6^ for *D. melanogaster*, *N*_*e*_ *s* ~ 3), such that the fixation probability of a newly arising advantageous mutation is very similar to that of a neutral allele. Such weak selection in *D. melanogaster* may not necessarily limit the frequency of hard sweeps, however, as it has been suggested that adaptation in *D. melanogaster* may be limited by current census population size rather than long-term *N*_*e*_ [54]. Secondly, the Elyashiv et al. [53] approach does not incorporate gene conversion, which may have a substantial impact on the effects of sweeps within genes [42]. Finally, their method overestimated the rate of deleterious mutations, though the authors suggested that this could be due to the presence of modes of adaptation other than hard sweeps in *D. melanogaster*.

## Haplotype structure can reveal both soft and incomplete selective sweeps

The extent to which adaptive evolution proceeds according to the hard sweep model is the subject of ongoing study. All of the approaches to infer the strength and tempo of adaptation we have discussed, with the exception of Coop and Ralph [33], have relied on either patterns of between-species substitution or the predictions made by hard sweep models. If adaptive change is limited by the supply of new mutations, hard sweeps must be the main mode of adaptive evolution. As described above, however, adaptation does not seem to be limited by the mutation rate, so perhaps alternative modes are common. The following section will describe how information carried in the distribution of haplotypes can be used to distinguish different forms of selective sweeps.

While a favoured allele is sweeping through a population, it carries with it linked variants on the same chromosome (Fig. 1). In the hypothetical case of a hard sweep arising from a single new beneficial mutation, with no further recombination or mutation, this will result in one haplotype coming to completely dominate the population. Although this situation is extreme, it serves as an example to highlight the fact that a lack of haplotype diversity, or, equivalently, an increase in LD between alleles at different sites, can be used as an indicator of the action of positive selection. In the case of soft sweeps, more than one haplotype may be elevated to a high frequency, and in the cases of incomplete and partial sweeps, a single haplotype may be at a higher frequency than expected under null models.

## Using haplotype structure to detect soft selective sweeps

The distribution of haplotypes at a locus has been analyzed to detect selection where adaptive evolution is very recent (for example [55–60]) and where it does not proceed according to the hard sweep model (for example [61–63]). Several test statistics have been proposed to analyze the distribution of haplotype frequencies in a sample (for descriptions of these see [64]). However, the power to detect selection decays quickly after a selective event ends [61]. There are several reasons for this, including the loss of ancestral haplotypes through genetic drift, recombination occurring before and after the fixation of an adaptive mutation shortening the haplotype generated by the sweep, and, finally, further mutation creating new haplotypes not associated with the initial sweep. The signatures present in the haplotype structure (for example a skew towards a small number of high frequency haplotypes) generated by positive selection persist for only ~ 0.01 *N*_*e*_ generations, which is an order of magnitude shorter than the persistence time of signatures in the site frequency spectrum [61, 65, 66].

Haplotype-based tests outperform diversity and site frequency spectrum-based tests at detecting soft sweeps. This is because, under the soft sweep model, several haplotypes may be carried to high frequency, resulting in characteristic signatures in a population’s haplotype structure, while leaving polymorphism less affected [61, 67]. There is now a sizeable amount of theoretical and empirical evidence suggesting that soft sweeps contribute to adaptive evolution in nature [66, 68]. For example, Garud et al. [62] introduced a set of haplotype-based statistics that together can detect both hard and soft sweeps, and discriminate between them. They applied their statistics to North American *D. melanogaster* and found evidence suggesting that soft sweeps are more common than hard sweeps. Similar results for a Zambian population were subsequently reported by Garud and Petrov [69]. However, soft sweeps arising from multiple de novo mutations require high beneficial mutation rates. In the case of soft sweeps from standing variation, even if alleles are segregating at appreciable frequencies in the population before the onset of selection, they may still be more likely to result in a hard sweep than a soft one (reviewed by [70]).

## Using haplotype structure to detect incomplete or partial selective sweeps

As is the case for soft sweeps, the signatures of both incomplete and partial selective sweeps left in polymorphism data are less clear than for hard sweeps (Fig. 1). For example, haplotype-based methods have revealed footprints of incomplete sweeps around certain alleles that are known to confer resistance to malaria [56]. If polygenic traits are the target of selection, partial sweeps may be common, because selection can bring about rapid evolution by acting on standing variation at multiple loci, affecting levels of diversity at linked neutral sites [67, 71]. A haplotype-based statistic introduced by Field et al. [63] called the singleton density score (‘SDS’) is able to detect very recent selection, including selection operating on polygenic traits. It quantifies the extent to which selection has distorted the genealogy of sampled haplotypes, as measured by the distribution of singleton mutations around ancestral and derived alleles at a focal locus. Field et al. provide evidence of selection on multiple polygenic traits, including height, in the ancestors of British people within the last 3000 years, suggesting that partial sweeps may be a common form of adaptive evolution. However, their study relied on published catalogues of genome-wide association study hits and > 3000 sequenced genomes, resources not available for most organisms. It remains to be seen whether these findings are general across different species groups. Finally, recent theoretical work by Jain and Stephan [72] suggests that the allele frequency shifts resulting from polygenic adaptation may be too subtle to be detected using common approaches, although this depends on the number of loci underlying quantitative traits. Indeed, quantitative traits can respond to selection when loci underlying the trait have *N*_*e*_ *s* < 1 [73]. Biologically grounded simulations using realistic trait architectures and selection regimes are likely necessary to determine how readily polygenic adaptation can be detected using population genomic data.

Patterns of LD can thus be used to infer the action of positive selection. Hard sweeps produce distinctive patterns of LD, but this information adds little for detecting hard sweeps when information from diversity and the site frequency spectrum is available [74], although it may be useful for distinguishing selection from demographic effects [75]. Haplotype information is useful, however, when selection is ongoing and/or it does not proceed according to the hard sweep model. One drawback of haplotype-based statistics is that they are often descriptive—although they provide a means for detecting sweeps, they do not provide a direct means for parameter estimation. An exception is the estimator of Messer and Neher [76], which is based on the frequency spectrum of haplotypes that arise during a sweep, and which may outperform diversity-based estimators of the strength of selection in some circumstances, although it requires a deep population sample (at least hundreds or thousands of sequences) to provide accurate estimates.

## Future directions: sweep modes and non-model organisms

Over the last ~ 30 years, much information about the action of natural selection has been leveraged from patterns of between-species substitution and within-species polymorphism. Researchers have accumulated evidence suggesting not only that adaptive evolution is frequent across a variety of species, but that it appears to be driven by strongly selected mutations. The application of recently developed tests and models to data from non-model organisms remains a challenge, however, since they variously require a population sample for very many individuals, a high quality reference genome and annotations, a genetic map and genome sequences of suitable outgroup species. Understanding the process of adaptive change in the genome across diverse taxa may therefore be challenging due to a lack of appropriate data.

A major challenge for understanding the forces of natural selection operating in the genome will be the incorporation of both soft and partial sweeps into theory and inference methods. The recent findings of Field et al. [63], Garud et al. [62] and Garud and Petrov [69] all suggest that both partial and soft sweeps may occur frequently. If modes of adaptation other than hard sweeps are common, current methods for inferring positive selection may result in systematically biased inferences. For example, a key parameter in the partial sweep model is the frequency that a beneficial mutation reaches before selection is ‘switched off’. As this critical frequency decreases, the inferred rate of sweeps increases over multiple orders of magnitude [33]. This example from theory, as well as the recent empirical results from population haplotype structure, should stimulate efforts to quantify the extent to which different sweep modes contribute to molecular evolution. To that end, Schrider and Kern have developed a machine learning approach [77] to classify region signatures of sweeps as either hard or soft. Application of their approach suggests that soft sweeps may be the dominant mode of adaptation in human evolution [78]. Estimating selection parameters based on the signatures of soft sweeps remains an open problem.

## Box 2 Glossary

DFE—the distribution of fitness effects for new mutations

Folded site frequency spectrum (folded SFS)—the distribution of minor allele frequencies in a sample of nucleotide sequences

Unfolded site frequency spectrum (unfolded SFS)—the distribution of derived allele frequencies in a sample of nucleotide sequences

*ɑ*—the proportion of substitutions that have been driven to fixation by positive selection, and not by other forces, such as drift

ω_*a*_—the rate of fixation of advantageous mutations relative to rate for neutral mutations

*N*_*e*_—effective population size

*s—*the absolute selection coefficient, the difference in fitness between homozygotes for wild-type alleles and homozygotes for mutant alleles (in diploids)

*N*_*e*_ *s—*the effective strength of selection, the strength of directional selection relative to random drift

LD—linkage disequilibrium, nonrandom associations of alleles at different loci
